# Are parasites “prudent” in space?

**DOI:** 10.1111/j.1461-0248.2010.01516.x

**Published:** 2010-10

**Authors:** Sébastien Lion, Mike Boots

**Affiliations:** 1 School of Biological Sciences, Royal Holloway University of LondonEgham TW20 0EX, UK; 2 Department of Animal and Plant Sciences, University of SheffieldSheffield S10 2TN, UK

**Keywords:** Epidemiology, kin selection, population dynamics, spatial structure, virulence

## Abstract

There has been a renewed controversy on the processes that determine evolution in spatially structured populations. Recent theoretical and empirical studies have suggested that parasites should be expected to be more “prudent” (less harmful and slower transmitting) when infection occurs locally. Using a novel approach based on spatial moment equations, we show that the evolution of parasites in spatially structured host populations is determined by the interplay of genetic and demographic spatial structuring, which in turn depends on the details of the ecological dynamics. This allows a detailed understanding of the roles of epidemiology, demography and network topology. Demographic turnover is needed for local interactions to select for prudence in the susceptible-infected models that have been the focus of previous studies. In diseases with little demographic turnover (as typical of many human diseases), we show that only parasites causing diseases with long-lived immunity are likely to be prudent in space. We further demonstrate why, at intermediate parasite dispersal, virulence can evolve to higher levels than predicted by non-spatial theory.

## Introduction

There is a striking variation in the life histories of infectious organisms. Some are highly infectious and virulent, whereas others are less virulent and chronic with longer infectious periods. Understanding the processes that determine these different parasite strategies of host exploitation is a key challenge in biology. It is increasingly clear that evolutionary theory is crucial to our understanding of parasite life histories since they are often shaped by the co-evolution of pathogens with their hosts (Anderson & May 1982; May & Anderson 1983; Ewald 1994; Van Baalen 1998; Dieckmann *et al*. 2002; Stearns & Koella 2007; Nesse & Stearns 2008; Restif 2009). By defining parasite fitness at an epidemiological level (Anderson & May 1991), one can investigate how different disease parameters (transmission, virulence, recovery) evolve and are influenced by the parasite's strategy of host exploitation (Anderson & May 1982). In particular, when would we expect “prudent” parasites with low infectivity and virulence and when would we expect to get fast transmitting deadly pathogens?

Recently, a body of work has suggested that “prudent” pathogens are selected for in viscous populatons, i.e. spatially structured host populations in which infections occur locally (Claessen & de Roos 1995; Rand *et al*. 1995; Boots & Sasaki 1999,^2000^; Haraguchi & Sasaki 2000; van Baalen 2002; O'Keefe & Antonovics 2002; Read & Keeling 2003; Kamo *et al*. 2006; Kamo & Boots 2006; Lion & van Baalen 2008). The majority of this theory uses the two-dimensional lattice to model spatial structure (Sato *et al*. 1994), but the same argument operates on social networks in which hosts are connected to their social group (van Baalen 2002). Given potential changes in the way in which populations mix, this is an important result that has recently received experimental support (Kerr *et al*. 2006; Boots & Mealor 2007). If populations become more mixed, fast-transmitting virulent pathogens are predicted to evolve. There are also clear parallels between the evolution of prudent parasites and the evolution of co-operation in viscous populations (e.g. parasite prudence can be interpreted as an altruistic trait; Frank 1996; van Baalen 2002; Lion & van Baalen 2008). It is therefore important, from both a practical and a fundamental theoretical evolutionary perspective, to understand how selection operates to produce these outcomes.

A recent paper by Wild *et al*. (2009) has shown convincingly that the evolution of lower virulence in spatially structured populations can be understood using an inclusive fitness argument, without invoking any new mechanism, in contrast with recent claims (Wilson & Wilson 2007). The analysis of Wild *et al*. (2009) did not aim to address the crucial issue of which life cycles are more conducive to the evolution of reduced virulence when parasite transmission is local. To answer this question, the details of the epidemiological and ecological dynamics need to be considered. Indeed, from the standpoint of evolutionary epidemiology, considering local infections adds two different levels of spatial structuring. At the epidemiological level, the distribution of individuals (e.g. susceptible vs. infected hosts) will change as a result of interactions being local, since for instance infected hosts will tend to be clustered. At the genetic level, population viscosity will also affect the spatial distribution of alleles. The probability that two neighbours are infected by the same parasitic strain is likely to be greater than in a mixed population. It is clear that evolution will depend crucially on the interplay between epidemiological and genetic spatial structuring (Box 1).

**Box 1 Demographic vs. genetic structuring**

The direction and speed of evolution in spatially structured environments are shaped by two levels of spatial structuring. *Demographic structuring* is the process by which local demographic events (such as birth, death, infection, dispersal and interactions with other individuals) lead to the spatial self-structuring of the population. *Genetic structuring* is the process by which population viscosity affects the spatial distribution of alleles. The method described in Box 2 and Appendix S1 allows us to decouple these two levels of spatial structuring, and effectively partitions the selective pressures on parasitic traits between demographic, epidemiological and genetic factors. As a result, the selection gradient is found to depend only on:

1. The *spatial distribution of individuals* of different types *in a monomorphic population* (for instance, the local densities of susceptible hosts experienced by infected hosts, *q*_*S*/*I*_, or the local density of empty sites around infected individuals, *q*_*o*/*I*_).
2. A measure of *genetic structuring*, which, in our simple genetic scenario, is simply the *relatednessr* between parasites infecting neighbouring hosts, as defined in kin-selection theory (e.g. for a rare mutant parasite in a dimorphic population, relatedness is *q*_*J*/*J*_, the probability that neighbours of a host infected by the mutant parasite are also infected by the mutant strain; Day & Taylor 1998; van Baalen & Rand 1998; Rousset 2004; Lion & Gandon 2009; Lion 2009).

Here, we compare the evolution of parasite strategies of host exploitation in spatially structured host populations, when parasites can infect locally or at a distance. We present a new analytical approach of understanding the relative importance and the interplay between genetic correlations and spatial ecological dynamics. Our approach generally gives a relatively simple series of expressions for inclusive fitness that analytically demonstrate how the outcome depends on a balance of genetic and demographic factors. In contrast with previous works, we are able to show how different assumptions about disease characteristics (with or without immunity), demography (host reproduction and mortality, and empty space) and network topology affect the evolutionary outcome. In particular, we explain when and why space does not always select for prudent parasites. We conclude by delineating the limits of the analytical approach we use, presenting some key results that cannot be explained easily using this method, and discussing open questions and wider applications of our approach.

## Analysis

In general, the characteristics of infectious diseases will be shaped by both the host and parasite. In this article, we shall assume that the parasite life-history traits depend only on the strategy of host exploitation of the parasite which in turn leads to physiological trade-offs between life-history traits, such as transmission and virulence. Therefore, we consider that transmission rate, recovery rate, disease-induced mortality are functions *β*(*e*), *γ*(*e*) and *α*(*e*) of host exploitation *e*. Such a trade-off between parasite life-history traits underpins much of the theoretical literature on virulence evolution (Anderson & May 1982; Ewald 1994; Dieckmann *et al*. 2002; Alizon *et al*. 2009), and is gaining increasing empirical support (reviewed in Alizon *et al*. 2009). The shape of the trade-off (between transmission and virulence, transmission and recovery, and so on) determines the evolutionary outcome (Anderson & May 1982; Frank 1996; van Baalen & Sabelis 1995; Alizon *et al*. 2009). Generally, transmission rate is assumed to saturate faster than virulence (or recovery) with exploitation creating a concave-down relationship between transmission and virulence (or recovery) that leads to an intermediate Evolutionary Stable Strategy (ESS).

### The susceptible-infective-susceptible (SIS) model with no demography

For simplicity, we start with the baseline SIS epidemiological model (Anderson & May 1991). Let us assume that hosts live on a network of sites. Each site can be occupied either by a susceptible host (*S*) or by an infected host (*I*). We are therefore assuming that the network is full, and that we can neglect host demography. This is the assumption of classical epidemiological models for the spread of a disease through a human population, and is likely to hold for many modern human diseases in developed countries.

Infected hosts can recover at rate *γ*, or transmit the disease to a susceptible host at rate *β*, either globally (with probability *P*) or locally (with probability 1−*P*). We assume that there is a cost of long-distance parasite dispersal (so that the propagule has survival probability *σ*). Furthermore, we assume a trade-off between transmission rate *β* and recovery rate *γ*. This spatial model is an extension of the model studied by Claessen & de Roos (1995).

Our aim is to determine under which conditions a rare mutant parasite can invade a host population infected by a resident parasitic strain (Box 2; Appendix S1). Let us consider a mutant parasite with life-history traits *β*_*J*_ and *γ*_*J*_ in a population infected by a resident parasite with traits *β*_*I*_ ≡ *β* and *γ*_*I*_ ≡ *γ*. The per-capita growth rate *λ*_*J*_ of the subpopulation of hosts infected by the mutant parasite can be written as (Boots & Sasaki 1999).

**Box 2 Approximating the selection gradient: biological assumptions**

Computing the selection gradient in models of evolutionary epidemiology is a difficult undertaking in general, but some insightful analytical expressions can be obtained assuming that mutations are rare and have small phenotypic effects.

*Separation of ecological and evolutionary time scales*. If mutations are rare, one can assume that evolution takes place at a slower pace than epidemiological dynamics. Then, the evolutionary success of a mutant parasitic strain can be measured by whether or not it can invade a monomorphic resident population when rare (Metz *et al*. 1992; Ferriére & Le Galliard 2001). This is a standard assumption of evolutionary game theory and adaptive dynamics (Metz *et al*. 1992; Dieckmann *et al*. 2002; Day & Gandon 2007).

*Weak selection*. Further analytical progress is possible if we assume that mutations have small phenotypic effects, as typically assumed in kin selection models (Day & Taylor 1998; Rousset 2004). This provides an expression for the selection gradient, which is a first-order approximation of the invasion fitness when selection is weak.

As shown in Appendix S1, these two assumptions on the mutation process yield to a general expression for the selection gradient Δ*S*: 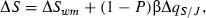 which separates a non-spatial component Δ*S*_*wm*_ and a spatial component (1 − *P*)*β*Δ*q*_*S*/*J*_, where *P* is the probability that infection occurs globally, *β* is the transmission rate in the resident population and Δ*q*_*S*/*J*_ measures the local competition of mutant parasites *J* for susceptible hosts *S*.

This general result shows that parasite evolution critically depends on the competitive term Δ*q*_*S*/*J*_. An exact expression, if obtainable at all, is currently beyond our reach, but it is possible to compute a first-order spatial approximation (see online supporting information). The core of the method has been discussed elsewhere (van Baalen & Rand 1998; Lion & Gandon 2009), and relies on deriving the dynamics of *pairs* of sites, then using moment closure approximation techniques such as the pair approximation (Matsuda *et al*. 1992; Sato *et al*. 1994; Rand 1999; van Baalen 2000). Note that the well-mixed limit can also be interpreted as a zeroth-order spatial approximation.

In summary, the method amounts to: (1) assuming that epidemiological dynamics occur on a faster time scale than evolutionary dynamics; (2) deriving a first-order approximation in the strength of selection (weak selection assumption) and (3) computing a first-order spatial approximation of the selection gradient. Clearly, these assumptions may be too strong for some infectious diseases, and we outline potential limitations and extensions of our approach in the discussion.

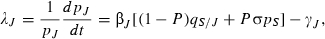
(1) where *p*_*J*_ is the global density of hosts infected by the mutant parasite, *p*_*S*_ is the global density of susceptible hosts and *q*_*S*/*J*_ is the local density of susceptible hosts in the neighbourhood of a host infected by the mutant parasite. Thus, *q*_*S*/*J*_ measures how many susceptible hosts are available on average for the local spread of the mutant parasite. Assuming that the mutant is rare, eqn 1 gives an implicit expression for the invasion fitness of the mutant parasite. Crucially, although the global density of susceptible hosts experienced by a rare mutant parasite is fixed by the resident parasite, the local density *q*_*S*/*J*_ depends on both the mutant and resident traits. This is because in a viscous population mutant parasites will tend to be clustered.

Assuming that selection is weak (mutations have relatively small phenotypic effects), we can take a further analytical step and determine the selection gradient, which is the first-order effect of selection on the invasion fitness *λ*_*J*_. Denoting by Δ*X* the first-order effect of the host exploitation strategy on *X*, we find, using *β*_*J*_ = *β* + Δ*β* and *γ*_*J*_ = *α* + Δ*γ*
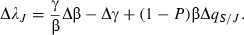
(2)

The first term is the benefit of increased transmission on parasite fitness, and is proportional to *γ*/*β*, which measures the availability of susceptible hosts in the resident population [at equilibrium, (1 − *P*)*q*_*S*/*J*_ + *Pσp*_*S*_ = *γ*/*β*]. The second term is the cost to the parasite fitness of recovery. The third term is the effect of mutant life-history traits on the local availability of susceptible individuals. If the mutant parasite's transmission rate is high, for instance, infection will be more frequent and locally the average number of susceptible neighbours may decrease. Therefore, Δ*q*_*S*/*J*_ measures the intensity of local competition for susceptible individuals.

A first observation is that, in a well-mixed population (*P* = 1), this local competition term vanishes. We then recover the classical result that, under a concave-down transmission-recovery trade-off, the ESS is the value that maximizes the ratio *β*/*γ* (Alizon 2008). The corollary of this result is that, in a population with limited parasite dispersal, departure from the prediction of non-spatial theory will be caused by the local competition of parasites for susceptible individuals. As a consequence, most of the analytical work used in this article will revolve around the computation of the competitive term Δ*q*_*S*/*J*_.

For the basic SIS model, we obtain the following expression (Appendix S3): 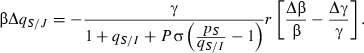
(3)

How can we interpret this expression? First, note that local competition for susceptible hosts depends on the marginal effects of the mutation on transmission (Δ*β*/*β*) and recovery (Δ*γ*/*γ*). Second, note the minus sign, which indicates that an increase in transmission and recovery have an opposite effect on local competition compared with the effects they have on parasite fitness in the well-mixed population. Third, we see that local competition for susceptible individuals is proportional to *r*, the between-host relatedness of parasites in neighbouring hosts. For a rare mutant parasite, *r* = *q*_*J*/*J*_, that is, the local density of mutant parasites experienced by a mutant parasite. The implication therefore is that Δ*q*_*S*/*J*_ can be interpreted as a measure of (between-host) kin competition. Thus, kin competition may be expected to be a crucial selective force shaping the evolution of parasite life-history traits.

In the basic SIS model, however, kin competition takes a very specific form, and using the method described in Appendix S1, we find that the resulting selection gradient is proportional to 
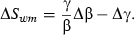
(4)

**Figure 1:**
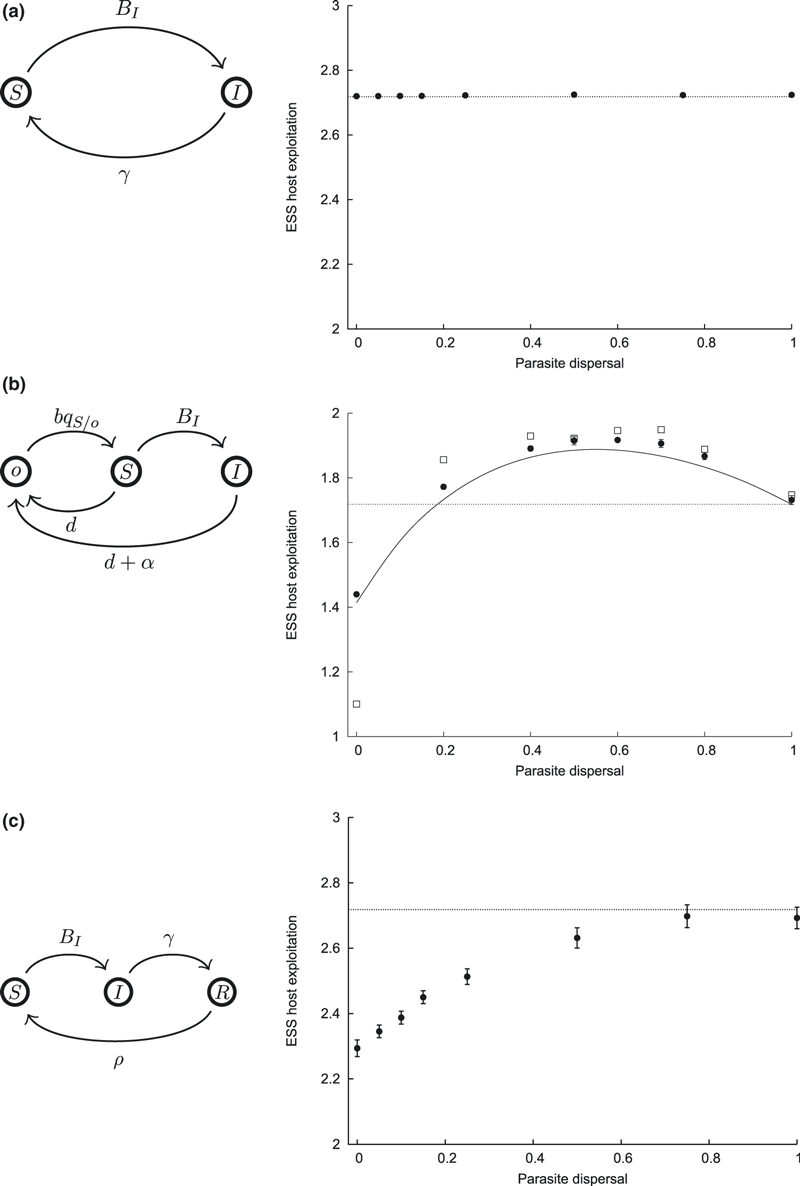
The evolutionarily stable host exploitation as a function of parasite dispersal *P*, for the (a) SIS model, (b) oSI model and (c) SIRS model. The schematics on the left-hand side give the average transition rates, where *B*_*I*_ = (1 − *P*)*βq*_*I*/*S*_ + *Pσβp*_*I*_ is the force of infection. On the right-hand side, the mean and standard deviation of eight runs of the stochastic process are presented. Mutations occured at rate 0.05. Mutation effects were drawn from a normal distribution with 0 mean and standard deviation 0.05. The mean equilibrium for each run was estimated as the average value of the trait between *t* = 20 000 and the simulation end time *t* = 35 000. Simulations were performed on a random network (circles) and a square lattice (squares), (b). The plain line in (b) gives the prediction of the first-order approximation of eqns 5 and 6. The dashed lines indicate the ES level of host exploitation predicted by non-spatial theory. Parameters: *b* = 8, *d* = 1, *n* = 4. The trade-off functions used are *β*(*e*) = 20 ln (1 + *e*) and *x*(*e*) = *e*, where *e* is the level of host exploitation and *x* is either recovery *γ* (a, c) or virulence *α* (b). Using other concave-down trade-off functions between transmission and virulence/recovery does not alter our qualitative results.

Therefore, the selective pressures on transmission and recovery do not depend on population viscosity or parasite dispersal, and the evolutionarily stable strategy is predicted to be the same as in a well-mixed population. This result provides an analytical underpinning to the results of Claessen & de Roos (1995), and is supported by extensive stochastic simulations (Fig. 1a). However, the prediction goes somewhat against the common expectation that population viscosity should favour prudent exploitation of the host population. In the following sections, we show that the interplay between population viscosity and ecological dynamics is a key point in determining the selective pressures on parasite life-history traits that lead to lower host exploitation.

## Demography matters: the susceptible-infected model with empty sites

Most studies for the evolution of spatially structured host–parasite interactions have considered the following scenario (Boots & Sasaki 1999, 2000; Haraguchi & Sasaki 2000; Kamo *et al*. 2006; Kamo & Boots 2006). Susceptible hosts can either die at rate *d*, or reproduce at rate *b* to a neighbouring site, in which case the offspring only survives if the site is empty (*o*). As before, infected hosts transmit the disease at rate *β*, either locally (with probability *P*) or globally (with probability 1 − *P*). Infected hosts can die at rate *d* + *α*, (natural death *d* and disease-induced mortality *α*) resulting in an empty site. In the absence of the disease, the host population goes to extinction if the reproduction rate *b* is below a critical value. As in the rest of the literature, we assume a trade-off between transmission *β* and virulence *α*.

A rare mutant parasite *J* will invade a resident population at endemic equilibrium if the selection gradient Δ*λ*_*J*_ is positive, where 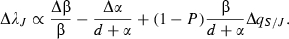
(5)

Non-spatial theory predicts the same evolutionary outcome in this model than in the previous SIS model, but in the SI model with empty sites (oSI), the effects of population viscosity encompassed in the competitive term Δ*q*_*S*/*J*_ are very different. It is shown in Appendix S5 that, when infections are local (*P* = 0) 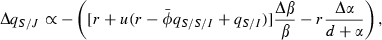
(6) where 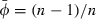, *q*_*S*/*SI*_ is the average local density of susceptible hosts experienced by a susceptible host with an infected host in its neighbourhood, and 
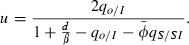


Again, we observe that the genetic structuring of the parasite population, as measured by the relatedness parameter *r*, plays a key role in shaping competition of parasites for susceptible hosts. But there is a striking difference between eqns 6 and 7: in the oSI model, a relative increase in transmission has an additional effect on competition compared with a relative increase in virulence.

The additional competitive term depends on the parameter *u*, which is proportional to the local density of empty sites experienced by an infected individual *q*_*o*/*I*_ (in the monomorphic population). When the density of empty sites is vanishingly small (*u* = 0), the competitive term in the oSI model collapses to the one in the SIS model. When *u* is not negligible, the evolutionary outcome depends on the sign of 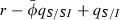. For a concave-down trade-off between transmission and virulence, the level of host exploitation will be lower for local (*P* = 0) than for global (*P* = 1) transmission if (Appendix S2): 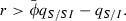
(7)

Note that eqn 7 separates the role of genetic structuring, through the relatedness parameter *r*, from the role of epidemiological structuring, through the difference 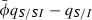, which measures the extent to which an infected individual can expect to infect susceptible hosts after it has infected its direct neighbours. It is not therefore just the proportion of susceptible hosts that are next to an infected that is crucial, but the subsequent availability of hosts. For local interactions to select for lower transmission and virulence, local relatedness needs to be high and susceptible hosts need to be relatively more available locally than at a distance. Extensive simulations suggest that in a purely viscous population, *r* should always be large enough for condition (7) to hold true. This implies that, in a population with host demography and empty sites, the evolutionary stable (ES) level of host exploitation in a purely viscous population (*P* = 0) should be lower than in a well-mixed population. Note that this effect is predicated on the existence of empty sites (or, equivalently, of host demography). If host fecundity is high, the local density *q*_*o*/*I*_ will vanish, and *u* = 0. Then, the evolutionary outcome is the same as in the SIS model. Any process that reduces the local density of empty sites *q*_*o*/*I*_ (such as the reproduction of infected individuals) should therefore select for higher levels of host exploitation, closer to what is predicted in a well-mixed population.

However, the simple prediction that parasites should be more prudent when infections are local is altered when we consider intermediate levels of dispersal (Kamo *et al*. 2006). The condition for host exploitation to be lower than in the well-mixed model then becomes 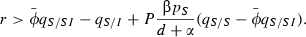
(8)

This expression differs from the previous condition through an additional term that relates to the benefit of long-distance dispersal, which is seen to be proportional to *P* and to *βp*_*S*_/(*d* + *α*), which is the *R*_0_ of the resident parasite in a well-mixed population. Therefore, increasing parasite dispersal will decrease the left-hand side (LHS) of eqn 8 through a decrease of genetic relatedness *r*, and also decrease the factor 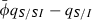, on the right-hand side (RHS) but it will also have a direct positive effect on the RHS. It is hard to see in which way the balance will be tipped from looking at eqn 8 alone, but numerical investigations show that virulence has a hump-shaped dependency on dispersal, so that virulence reaches a maximum at an intermediate level of dispersal (Fig. 1). This was first pointed out by Kamo *et al*. (2006). Interestingly, for a wide range of parasite dispersal, virulence evolves to higher levels than in a well-mixed population. Thus, although low levels of dispersal do select for lower levels of host exploitation, virulence can peak at intermediate values of dispersal. Note that the value of *P* at which the RHS and LHS of eqn 8 become equal yields an estimate for the critical value *P*_*c*_ of parasite dispersal above which virulence is predicted to be higher than in a well-mixed population.

Figure 1b shows that our analysis accurately predicts the pattern observed in stochastic simulations, both on a random regular network (a network in which each site is connected to *n* randomly chosen sites) and on a square lattice. In the latter case, our approximation fails for very low values of parasite dispersal, but performs well in the range of parasite dispersal for which virulence is higher than in a well-mixed population. In particular, the value of *P*_*c*_ does not seem to be very different on the two networks.

### Epidemiology matters: the role of host immunity (SIRS model)

The oSI model is a good description of many wild-life diseases which strongly affect the demography of the host population. For most human diseases, however, disease-induced mortality rates are generally too low to affect the dynamics of the population, and as a result, the epidemiological dynamics occur on a much faster time scale than the dynamics of the host population (Anderson & May 1991). For human diseases, we may therefore assume that host population is fairly constant. The basic SIS model that we have already analysed is the simplest model satisfying this assumption, and suggests that space is not important to human diseases without long-lasting immunity. Many important human diseases do have long-lived immune memory and the classic extension to the SIS model is the susceptible-infective-recovered-susceptible (SIRS) model (Anderson & May 1991), in which infected hosts can recover and enter an immune class (*R*). Recovered individuals lose their immunity at rate *ρ*. This model was studied in a spatial context by van Baalen (2002), in the limit when infections are only local.

Importantly, the selection gradient in the SIRS model is also given by eqn 6. This entails that, once more, the ESS in a well-mixed population is the same as in the SIS model, that is, it is determined by the trade-off between the transmission rate *β* and the recovery rate *γ* (Alizon 2008). Things are different in a viscous population however, because local competition for susceptible hosts takes a different form in the SIRS model compared with the SIS model.

Let us assume that transmission occurs locally only (*P* = 0). It is shown in Appendix S4 that the local competition term is 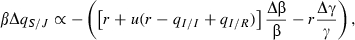
(9) where *u* = 2*γ*/*ρ*. Note the structural similarity between eqns 6 and 9. As in the oSI model, we see that there is an additional competitive term in the SIRS model compared with the SIS model. In the SIRS model, a relative increase in transmission has an additional effect on competition compared with a relative increase in recovery.

The additional competitive term depends on the parameter *u*, which is proportional to the ratio between infected and recovered individuals *γ*/*ρ* = *p*_*R*_/*p*_*I*_, which can also be expressed in terms of local densities as *q*_*R*/*I*_/*q*_*I*/*R*_. When *u* = 0, which occurs when the recovered class is short-lived (*γ*→0, or equivalently *p*_*R*_ and *q*_*R*/*I*_→0), the competitive term in the SIRS model collapses to the one in the SIS model. When *u* is not negligible, the evolutionary outcome depends on the sign of *r* − *q*_*I*/*I*_ + *q*_*I*/*R*_. Extensive numerical exploration suggests that 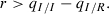
(10)

This implies that the weighing factor in front of Δ*β*/*β* is larger than the weighing favour in front of Δ*α*/*α*. Therefore, under a concave-down trade-off between virulence and transmission, the ESS level of host exploitation should be lower in a viscous population than in a well-mixed population (Appendix S2), as confirmed by stochastic simulations (Fig. 1c). As previously, eqn 10 separates the role of genetic structuring, through the relatedness parameter *r*, from the role of epidemiological structuring, through the difference *q*_*I*/*I*_−*q*_*I*/*R*_, which measures whether infected hosts experience a higher number of infected neighbours than recovered hosts. Note also that this result is predicated on the existence of an immune class. In the limit where loss of immunity is very fast (*ρ*→∞, i.e. *u*→0), the system and evolutionary predictions reduce to the SIS model. Therefore, if host immunity is short-lived, or if it is imperfect, the level of host exploitation should increase and tend towards the level of host exploitation predicted by non-spatial theory.

### The role of network topology

The expressions for the selection gradient obtained in the three scenarios above rely on an approximation of spatial structure. In effect, we assume that we can neglect the selective effects of spatial configurations such as interactions between triplets of individuals, and only retain the selective pressures due to pairwise interactions. It is well known that the quantitative accuracy of such approximations will be sensitive to the topology of the spatial or social network (Rand 1999). However, what about qualitative predictions?

**Figure 2:**
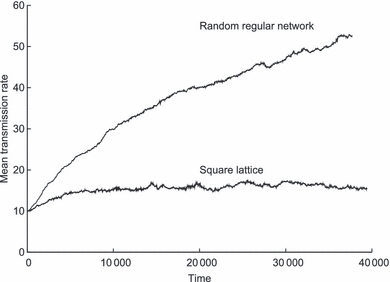
Evolution of tranmission rate in the oSI model in the absence of a trade-off with virulence on a random regular network and a square lattice, starting from transmission rate *β* = 10. Parameters: *α* = 0 (no virulence). Mutation process and other parameters as in Fig. 1.

In the oSI model, for instance, our first-order approximation predicts that transmission rate should be maximal in the absence of a trade-off with virulence (results not shown). This prediction is borne out by simulations on a random regular network, which is expected because the selective effects of higher order spatial correlations on this type of network is often negligible. However, on a lattice, the transmission rate has been shown to evolve to a finite value in the absence of a trade-off (Haraguchi & Sasaki 2000). Figure 2 illustrates this qualitative effect of network topology on the evolution of parasite transmission rate.

The failure of our first-order approach indicates that we need to take into account larger-scale spatial correlations to understand the evolutionary dynamics of transmission on a lattice. Interestingly, when a trade-off between transmission and virulence is assumed, the first-order approximation of the selection gradient predicts the outcome well, both qualitatively and quantitatively. Our interpretation is that, under a trade-off, the evolution of parasite traits are constrained in a domain where large-scale correlations do not play a significant evolutionary role. In the absence of a trade-off, however, the transmission rate can evolve to values sufficiently high for large-scale correlations to become potentially important, in which case the evolutionary effect will remain small on a random network but become important on a square lattice.

A reason for this is that evolution towards intermediate transmission rate on a lattice seems to be due to local extinctions of clusters of hosts infected by a single parasite clone. In other words, a higher transmission rate is selected for until a critical value is reached beyond which any further increase in transmission will cause the local cluster of hosts to be wiped out very rapidly. This local “tragedy of the commons” can only be fully captured if the process is modelled at the relevant scale, and it is clear that the pair level on which we focus does not allow this. Indeed, it may actually be very hard to quantify this type of mechanism with pairs or triples only, and alternative approaches may be more appropriate. A possible method would be to study an alternate patch model, which can be analysed by means of simulations (Rand *et al*. 1995), inclusive fitness analyses (Wild *et al*. 2009) or multilevel selection analyses (see Kelly 1994, for a related example). But the key point is that the patch model considered has to be based on the ecological dynamics of the lattice model (Rand *et al*. 1995). In other words, the scale at which patches are defined must represent the scale of the process on the lattice, and most importantly, the size and spatial scale of patches must not be fixed parameters, but dynamical variables affected by the trait. This may prove to be a difficult mathematical challenge.

Although this may seem a rather technical point, it has also some important biological consequences, as it implies that, under some life-history assumptions, the underlying structure of the social network on which the disease spreads may lead to qualitatively different predictions for the evolution of host exploitation.

## Discussion

Spatial structuring is an important component of the feedback loop between ecological and evolutionary dynamics (Lion & van Baalen 2008). Our approach shows that the evolution of “prudent” strategies of host exploitation in parasites strongly depends on how demographic and genetic spatial structuring interplay. The main message is that reduced levels of host exploitation in spatially structured host–parasite interactions evolve because population viscosity tends to locally increase the competition for susceptible individuals. Our analytical approach allows us to show that this local competition term depends on both genetic factors (such as between-host relatedness between parasitic strains) and demographic factors (such as habitat saturation around infected individuals or epidemiological structuring).

Our analysis shows that different epidemiological and ecological scenarios (SIS, SIRS and oSI models) lead to fundamentally different interactions between parasite dispersal, epidemiological structuring and genetic structuring. Hence, the evolutionary outcome will depend strongly on the details of the particular host–parasite interactions. For the SIS model under concave-down trade-offs between parasite transmission and recovery, we find that parasite dispersal has no effect on the ES level of host exploitation, and that relatedness only affects the speed at which this level is reached. For mild pathogens that do not regulate the host population, the spatial SIRS model predicts a monotonous increase in virulence as populations become well-mixed, and therefore the predictions of non-spatial theory can be seen as a worst-case scenario. For pathogens that cause significant host mortality, the outcome depends on the shape of the trade-off between transmission and virulence. Our analysis predicts either a monotonous increase (linear trade-off; Boots & Sasaki 1999) or a hump-shaped relationship [the standard saturating (concave-down) trade-off; Kamo *et al*. 2006]. In the latter case, the non-spatial theory does not yield a worst-case scenario anymore, and for some life history assumptions, the predicted level of virulence is actually higher than predicted by non-spatial theory for a large range of parasite dispersal. In other words, although parasites are indeed more “prudent” in a purely viscous population with only local infections, things are more complicated at intermediate parasite dispersal, which is an effect first noted by Kamo *et al*. (2006). That parasites need not always be prudent in space was also noted by Boots *et al*. (2004) and Read & Keeling (2006).

The prediction that virulence may peak at intermediate parasite dispersal could have implications for the management of human and animal diseases. Indeed, it is increasingly recognized that modern contemporary societies and trade routes exhibit a ‘small-world effect’, with interactions occuring at both a local and a global scales as in our model (Watts & Strogatz 1998; Boots & Sasaki 1999; Brockmann *et al*. 2006). It is therefore of critical importance to undestand how changes in the contact patterns of human or animal interactions and pathogen transmission may affect the evolution of diseases. Our analysis shows that the answer will depend on the details of disease life history, and further studies are needed to investigate in detail how different assumptions on host demography and epidemiology affect these predictions.

A key insight from our results is that ecology is crucial to evolution in space, because ecological interactions shape the demographic, epidemiological and genetic structure of the population. At the epidemiological level, the details of host and parasite life histories affect the spatial distribution of susceptible and infected individuals. At the genetic level, they affect the spatial distribution of parasitic strains among infected hosts. When selection is weak, this genetic structure can be captured using between-host relatedness. It is important to emphasize that relatedness is an ecological variable, and not a fixed parameter of the species. In general, relatedness depends on parasite and host dispersal, because these processes affect the probability that genes between two individuals are shared by a common ancestor (Rousset 2004).

It is not surpising that between-host relatedness emerges as a crucial predictor of reduced host exploitation. It has long been recognized that reduced host exploitation can be seen as an altruistic trait: from the point of view of a parasite, reducing one's transmission has a fitness cost, but may yield fitness benefits to neighbouring parasites competing for the same susceptible hosts (van Baalen 2002; Lion & van Baalen 2008). In a viscous population, the cost of local competition for susceptible hosts is higher because parasites tend to compete with related parasites. Hence, it should pay to be “prudent”. In this study, we show, however, that higher relatedness need not always result in a monotonous decrease in host exploitation. The outcome depends on the details of the ecological interactions.

That kin selection theory is implicitly rooted in spatial ecology is clear from Hamilton's (1964) seminal paper, but often overlooked. Many models of kin selection assume for instance that population density is constant (but see Rousset & Ronce (2004)). Because the selective pressures on host exploitation will depend in a subtle way on the dynamics of host and parasite densities, the study of host–parasite interactions forms a key area where we need to put ecology back into kin selection theory. The approach we propose is a step in that direction by providing an inclusive fitness argument explicitly derived from the epidemiological dynamics. By partitioning the selection gradient between genetic and demographic componennts, our approach makes clear connections with ecology and population genetics, as well as kin selection theory through the relatedness parameter. Relative to previous models (Wild *et al*. 2009), our method yields compact mathematical expressions for the selection gradient which allow analytical inferences on how selective pressures vary in response to traits such as host fecundity or parasite dispersal. It should be noted that, although our approach naturally lends itself to a kin selection interpretation, it would also be possible to adopt a multilevel selection perspective (Bijma & Wade 2008; Lion & van Baalen 2008). However, because our model does not assume a fixed group structure, a multilevel selection analysis is likely to be more unwieldly in this context.

Another potentially important advantage of our modelling approach is that the main determinants of the evolutionary outcome can in principle be measured in natural systems, or experimentally controlled and manipulated in the laboratory. In particular, the measure of relatedness we use can be linked to the spatial distribution of different parasitic strains, but also to measures of genealogical descent (Rousset 2004; Lion 2009). Genetic typing of infections may allow at least in principle for this data to be collected. The role of demographic and epidemiological structuring could also be quantified and manipulated by tracking the contacts of individuals, or varying the saturation or viscosity of the habitat (Kerr *et al*. 2006; Boots & Mealor 2007; Kümmerli *et al*. 2009).

Our first-order approximation allows us to understand the evolution of parasite traits in spatially structured host populations for a variety of epidemiological scenarios, but some particular phenomena may require additional analyses. For instance, as explained previously, our analysis does not allow us to explain the observed evolution of intermediate transmission in the absence of a trade-off with virulence on a lattice (Haraguchi & Sasaki 2000). This implies that, for this particular eco-evolutionary process, large-scale interactions, which we neglect in our analysis, need to be taken into account. Similar results are expected in models where large-scale spatial patterns are determinant, such as the SIRS model with fixed infectious and immune periods studied by van Ballegooijen & Boerlijst (2004). Despite this limitation, our inclusive fitness approach is still successful in predicting complex patterns, such as the hump-shaped relationship of virulence with dispersal observed in the oSI model (even on the square lattice) even though there is significant spatial clustering in the population. The reason (and important lesson) is that complex ecological dynamics do not necessarily imply that a complex evolutionary mechanism underpins the observed evolutionary dynamics.

To obtain our analytical results, we used an invasion analysis and assumed that mutations are rare and have small phenotypic effects. The robustness of our approximation was then checked against stochastic simulations in which the parasite population is allowed to be polymorphic and mutation to occur at higher rates than assumed in our approximation. However, the simulations still assume that mutational variance and mutation rates are low. Clearly, this is a strong assumption for many infectious diseases, as many pathogens (especially RNA viruses) are characterized by high mutation rates and mutations of large effect (Duffy *et al*. 2008). In this situation when selection is strong and the time scales between epidemiological and evolutionary dynamics overlap, an approach tying together population genetics and evolutionary epidemiology could be more appropriate (Day & Gandon (2007); Lion and Gandon, Unpublished).

From an applied perspective, we think that the framework we present here is a fruitful way to investigate the evolutionary consequences of host–parasite co-evolution and of changes in the structure of human societies. In particular, it provides an interesting modelling approach in which to study the impact of public health policies, such as vaccination strategies, on the evolution of parasites. To address more realistic genetic, ecological and epidemiological scenarios, it will be necessary to relax some of our assumptions (allowing for instance higher mutation rates, larger phenotypic effects or heterogeneity in the host population). This should not affect, however, the main message of our study: ecology really matters.

## SUPPORTING INFORMATION

**Appendix S1** Deriving the selection gradient.

**Appendix S2** Finding the condition for parasite prudence under a concave-down trade-off.

**Appendix S3** The baseline model: SIS model.

**Appendix S4** The role of host immunity: SIRS model.

**Appendix S5** The role of host demography: oSI model.

As a service to our authors and readers, this journal provides supporting information supplied by the authors. Such materials are peer-reviewed and may be re-organized for online delivery, but are not copy-edited or typeset. Technical support issues arising from supporting information (other than missing files) should be addressed to the authors.
