# Supplementary material for: Are parasites “prudent” in space?
Source: Ecol Lett. 2010 Oct;13(10):1245–55. doi: 10.1111/j.1461-0248.2010.01516.x (PMC3070161; doi:10.1111/j.1461-0248.2010.01516.x)
Supplement: Supplementary file 1 [file ele0013-1245-SD1.pdf]

Supporting information for *Are parasites "prudent" in space?*

Sébastien Lion and Mike Boots

July 30, 2010

In this appendix, we sketch the important steps of the derivation of the selection gradient in the three models we use. Further details about the method used can be found in van Baalen and Rand (1998), Lion and Gandon (2009), and Lion (2009).

S1 Deriving the selection gradient

In a host population infected by a single parasitic strain, the expected dynamics of the density of infected hosts p_I follows

$$\frac{dp_I}{dt} = [\beta_I((1-P)q_{S/I} + P\sigma p_S) - \delta_I]p_I$$

where β_I is the transmission rate, P the probability that infection occurs globally (with propagule survival σ), and δ_I is the rate at which individuals are removed from the infected class (i.e. $\delta_I = \gamma_I$ in the SIS and SIRS model, and $\delta_I = d + \alpha_I$ in the SI model with empty sites). At equilibrium, we find that the global density of susceptible hosts p_S and the local density of susceptible hosts $q_{S/I}$ are linked through the relationship

$$(1-P)q_{S/I} + P\sigma p_S = \frac{\delta_I}{\beta_I}. \tag{1}$$

Assuming that ecology takes place over a fast time-scale (i.e. mutations are rare), one can assume that a mutant parasite will arise in a population at ecological equilibrium. Its per-capita growth rate will be (Boots and Sasaki, 1999)

$$\lambda_J = \beta_J((1-P)q_{S/J} + P\sigma p_S) - \delta_J$$

where β_J is the transmission rate for the mutant parasite, and δ_J is the rate at which individuals are removed from the infected class. The total infection strength depends on the global density of susceptible hosts p_S and on the local density $q_{S/J}$. The latter measures how many susceptible hosts a mutant parasite has in its neighbourhood on average.

Assuming further that selection is weak (i.e. mutations have small phenotypic effects, so that the mutant trait is close to the resident trait), the selection gradient is given by the first-order approximation of λ_J. We obtain

$$\Delta\lambda_J = \Delta\beta_J((1-P)q_{S/I} + P\sigma p_S) - \Delta\delta_J + (1-P)\beta_I\Delta q_{S/J}$$

where Δ is a short notation for $\partial/\partial e'$ evaluated at $e' = e$. Using equation (1), this can be rewritten as

$$\Delta\lambda_J = \frac{\delta_I}{\beta_I}\Delta\beta_J - \Delta\delta_J + (1-P)\beta_I\Delta q_{S/J}. \tag{2}$$

Hence, the selection gradient only depends on the resident parasite's traits β_I and δ_I, on the marginal effects of the mutation on the transmission rate, on the death (or recovery) rate, and on the local density of susceptible hosts experienced by a mutant parasite.

This allows the selection gradient to be split into a non-spatial component $\Delta S_{wm} = \delta_I/\beta_I\Delta\beta - \Delta\delta$, and a spatial component $(1-P)\beta_I\Delta q_{S/J}$. In a well-mixed population ($P = 1$), the second term on

the right-hand side vanishes, and we recover the classical result that evolution should proceed as to maximise the ratio β/δ. In a spatially structured population, however, we need to compute the term $\Delta q_{S/J}$, which measures the local competition of mutant parasites for susceptible hosts. A first-order spatial approximation of this competitive term is derived belowfor the three epidemiological models we consider.

S2 Finding the condition for parasite prudence under a concave-down trade-off

Generally, we obtain expressions for $\Delta q_{S/J}$ of the form

$$\Delta q_{S/J} = -\frac{1}{\kappa}\frac{\delta}{\beta}\left[(r+uF)\frac{\Delta\beta}{\beta} - r\frac{\Delta\delta}{\delta}\right]$$

where $\kappa > 1$. Together with equation (2), this implies that any candidate ESS is a solution of

$$\frac{(\Delta\beta/\beta)}{(\Delta\delta/\delta)} = \frac{\kappa-(1-P)r}{\kappa-(1-P)r-(1-P)uF}, \tag{3}$$

all terms in the equation being evaluated at $e' = e$ (i.e. in the neutral model). When $P = 1$, this collapses to

$$\frac{\Delta\beta}{\Delta\delta} = \frac{\beta}{\delta}$$

which is the marginal value theorem. This implies that the ESS is the value of e at which the ratio β/δ is maximised. Using the notations $\beta = f(x)$ and $\delta = x$ for notational simplicity, this means that when dispersal is global, the ESS is the zero of $g(x) = xf'(x) - f(x)$. Now assume that the function is such behaved that such a zero exists and is unique. Biologically meaningful trade-off function will usually satisfy this criterion, but the function will need to be concave-down and monotonously increasing. Let us denote x_0 the zero of $g(x)$. It is straightforward to show that $g(x)$ is a monotonously decreasing function of x which is positive when $x < x_0$ and negative when $x > x_0$.

Returning to equation (3), this implies that the ESS in the spatial model will be lower than in the non-spatial model when the left-hand side is larger than 1, or equivalently, when $uF > 0$. This allow us to derive conditions (7), (8) and (10) in the main text without computing exactly the candidate ESS.

S3 The baseline model: SIS model

S3.1 Monomorphic population

Here, we show how to compute the equilibrium density $q_{S/SI}$, which proves useful in the simplification of the expression for the selection gradient in section S3.2. In the monomorphic population, the dynamics of pairs SI and II are given by

$$\begin{aligned}
\frac{dp_{SI}}{dt} &= \gamma p_{II} + p_{SS}((1-P)\beta\bar{\phi}q_{I/SS} + P\sigma\beta p_I) - p_{SI}(\gamma + (1-P)\beta(\phi+\bar{\phi}q_{I/SI}) + P\sigma\beta p_I) \\
\frac{dp_{II}}{dt} &= -2\gamma p_{II} + 2p_{SI}\beta((1-P)(\phi+\bar{\phi}q_{I/SI}) + P\sigma p_I),
\end{aligned}$$

where $\phi = 1/n$ is the inverse of the number of neighbours of each site on the network, and $\bar{\phi} = 1-\phi$. Adding the second equation and twice the first equation yields at equilibrium

$$0 = 2p_{SS}((1-P)\beta\bar{\phi}q_{I/SS} + P\sigma\beta p_I) - 2\gamma p_{SI}.$$

Using the fact that $p_{SS}q_{I/SS} = q_{S/SI}p_{SI}$ and $p_{SS} = p_S - p_{SI}$, we can rearrange this equation and obtain an exact expression for $q_{S/SI}$ as a function of $q_{S/I}$ and p_S. Using the fact that at equilibrium

$$(1-P)q_{S/I} + P\sigma p_S = \frac{\gamma}{\beta}, \tag{4}$$

we can further express the equilibrium density $q_{S/SI}$ in the monomorphic population in function of $q_{S/I}$ only. We find that, at equilibrium,

$$\bar{\phi} q_{S/SI} = q_{S/I}. \tag{5}$$

S3.2 Dimorphic population

The per-capita growth rates of I and J individuals are

$$\lambda_I = (1-P)\beta_I q_{S/I} + P\sigma\beta_I p_S - \gamma_I$$

and

$$\lambda_J = (1-P)\beta_J q_{S/J} + P\sigma\beta_J p_S - \gamma_J.$$

In the neutral model ($\beta_I = \beta_J = \beta$, $\gamma_I = \gamma_J = \gamma$), it follows that at equilibrium $q_{S/J} = q_{S/I}$. In the model with selection, if the mutant is rare and the resident population is at equilibrium, we have $\lambda_I = 0$, and we can Taylor-expand the invasion fitness λ_J around the neutral point. This gives

$$\Delta\lambda_J = [(1-P)q_{S/I} + P\sigma p_S]\Delta\beta + (1-P)\beta\Delta q_{S/J} - \Delta\gamma,$$

which can be futher simplified using equation (4) as

$$\Delta\lambda_J = \frac{\gamma}{\beta}\Delta\beta + (1-P)\beta\Delta q_{S/J} - \Delta\gamma. \tag{6}$$

Note that although the global density p_S is fixed by the resident trait, the local density $q_{S/J}$ will depend on the reproductive effort of mutants.

Equipped with this first expression for invasion fitness, we will now derive the expression of $\Delta q_{S/J}$. Let $\boldsymbol{p}$ be the vector of pair densities $\begin{pmatrix} p_{SJ} & p_{IJ} & p_{JJ} \end{pmatrix}^T$. The dynamics of $\boldsymbol{p}$ is given by

$$\frac{d\boldsymbol{p}}{dt} = \boldsymbol{M}\boldsymbol{p},$$

where $\boldsymbol{M} = \boldsymbol{M}_0 + P\sigma\boldsymbol{M}_1$. From van Baalen and Rand (1998) and Lion and Gandon (2009), we know that

$$\boldsymbol{M}_0 = \begin{pmatrix} -(1-P)\beta_J(\phi + \bar{\phi}q_{J/SJ} - \bar{\phi}q_{S/SJ}) - (1-P)\beta_I\bar{\phi}q_{I/SJ} - \gamma_J & \gamma_I & \gamma_J \\ (1-P)(\beta_I + \beta_J)\bar{\phi}q_{I/SJ} & -\gamma_I - \gamma_J & 0 \\ 2(1-P)\beta_J(\phi + \bar{\phi}q_{J/SJ}) & 0 & -2\gamma_J \end{pmatrix}.$$

The mortality terms are simple to understand because mortality is density-independent in this model. Therefore, a term of the form $\gamma_I p_{IJ}$ indicate the contribution to the dynamics of the death of a I individual in a IJ pair. For reproduction events, some further explanation is needed. First, the matrix $\boldsymbol{M}_0$ only collects the contributions of *local* reproduction events, hence the factor $(1-P)$. Second, the first column of $\boldsymbol{M}_0$ derives from a bookkeeping of all possible reproductive events that may affect a pair SJ. Consider for instance the transition $SJ \to JJ$, which yields the term in the lower left-hand side corner of the matrix. Then, the susceptible individual can be infected either by the infected individual in the pair (at rate $\beta_J/n = \phi\beta_J$) or by a J individual connected to the susceptible individual in the SJ pair, which occurs at rate $(n-1)/nq_{J/SJ}\beta_J$. The factor 2 on the last line of $\boldsymbol{M}_0$ comes from the fact that pairs are counted in both directions (Rand, 1999), hence pairs JJ are counted twice.

Computing $\boldsymbol{M}_1$ is a bit more complex, but we obtain the following expression

$$\boldsymbol{M}_1 = \begin{pmatrix} -(\beta_J p_J + \beta_I p_I) + \frac{q_{S/S}}{q_{S/J}}p_S\beta_J & 0 & 0 \\ \frac{q_{S/I}}{q_{S/J}}\beta_J p_I + \beta_I p_i & 0 & 0 \\ 2p_J\beta_J & 0 & 0 \end{pmatrix}.$$

The term on the first row gives the rate at which a pair SJ is altered by long-distance reproduction events; either the pair is destroyed when a random I or J individual infects the susceptible individual in

the pair, or it is created from a SS pair through long-range reproduction of a mutant. This affects the dynamics of SJ pairs by a term $\beta_J p_J p_{SS}$, which can be rewritten because $p_J = p_{SJ}/q_{S/J}$. The term on the second row gives the rate at which a IJ pair is created when a random resident individual infects the susceptible individual in a SJ pair, or when a random mutant individual infects the susceptible individual in a SI pair. The contribution to the dynamics is then $\beta_J p_J p_{SI} + \beta_I p_I p_{SJ}$ which can be rewritten using the same trick as above. Finally, the term in the third row gives the rate at which pairs JJ are created through long-distance infection by mutants of the susceptible individual in a SJ pair. Assuming the mutant is rare, the expression of M_1 can be further simplified using $p_J \approx 0$

$$\boldsymbol{M}_1 = \begin{pmatrix} -\beta_I p_I + \frac{q_{S/S}}{q_{S/J}} p_S \beta_J & 0 & 0 \\ \frac{q_{S/I}}{q_{S/J}} \beta_J p_I + \beta_I p_I & 0 & 0 \\ 0 & 0 & 0 \end{pmatrix}.$$

At neutrality ($\beta_J = \beta_I = \beta$, $\gamma_J = \gamma_I = \gamma$, and $q_{S/I} = q_{S/J}$), the expression of the invasion matrix $\boldsymbol{M}$ can be simplified, and we can compute the left and right eigenvector of the neutral matrix associated with eigenvalue 0. For the left eigenvector, we find $\boldsymbol{v} = \begin{pmatrix} 2 & 1 & 1 \end{pmatrix}$, and we know the right eigenvector is the vector $\boldsymbol{u} = \begin{pmatrix} q_{S/J} & q_{I/J} & q_{J/J} \end{pmatrix}^T$ (van Baalen and Rand, 1998; Lion and van Baalen, 2009). Further algebra yields

$$\boldsymbol{u} = \begin{pmatrix} q_{S/I} & \frac{\beta q_{S/I}}{\gamma}(P\sigma p_I + (1-P)\bar{\phi} q_{I/SJ}) & \frac{\beta q_{S/I}}{\gamma}(1-P)(\phi + \bar{\phi} q_{J/SJ}) \end{pmatrix}^T.$$

Note that this gives in particular an equation for the nearest-neighbour relatedness $r = q_{J/J}$ (Lion and Gandon, 2009; Lion, 2009) as a function of $q_{J/SJ}$.

Assuming that the local densities equilibrate on a fast time scale compared to the global density of mutants, the invasion matrix $\boldsymbol{M}$ can be approximated as a constant matrix whose dominant eigenvalue is the per-capita growth rate of the mutant when rare, that is, the invasion fitness (van Baalen and Rand, 1998; Ferrière and Le Galliard, 2001; Lion and van Baalen, 2009). The selection gradient is then given by

$$\Delta\lambda_M = \frac{\boldsymbol{v}\,\Delta\boldsymbol{M}\,\boldsymbol{u}}{\boldsymbol{v}\boldsymbol{u}},$$

where $\boldsymbol{\Delta M} = \boldsymbol{\Delta M_0} + P\gamma\boldsymbol{\Delta M_1}$ is the first-order effect of selection on the invasion matrix.

Assuming the mutant is rare and the resident population is on the monomorphic attractor, the only variables that will be affected by a change in reproductive effort are β_J, γ_J, $q_{S/J}$ and the triple local densities $q_{i/SJ}$. Using the expressions for $\boldsymbol{u}$, $\boldsymbol{v}$ and $\boldsymbol{M}$, we obtain after some rearrangements and simplifications an expression for $\Delta\lambda_J$ as a function of $\Delta\beta$, $\Delta\gamma$, $\Delta q_{S/J}$ and $\Delta q_{S/SJ}$ (note that $\Delta q_{S/SJ} = -\Delta q_{I/SJ} - \Delta q_{J/SJ}$). The coefficients of the Δ terms are all evaluated in the neutral model.

Along with equation (6), this gives a system of two equations with two unknowns $\Delta\lambda_J$ and $\Delta q_{S/J}$. Solving the system yields an expression for $\Delta q_{S/J}$ that depends on $q_{S/I}$, $p_S = 1 - p_I$, $q_{S/SI}$, $q_{I/SJ}$ and $q_{J/SJ}$. Further simplifications are possible, because p_S and $q_{S/SI}$ can be expressed in terms of $q_{S/I}$ (equations (4) and (5)), because $q_{I/SJ} = 1 - q_{J/SJ} - q_{S/SJ} = 1 - q_{J/SJ} - q_{S/SI}$, and $q_{J/SJ}$ can be expressed in terms of nearest-neighbour relatedness $r \equiv q_{J/J}$ using the expressions of the eigenvector $\boldsymbol{u}$. Putting everything together we finally obtain

$$\beta\Delta q_{S/J} = -\frac{\gamma}{1 + q_{S/I} + P\sigma\left(\frac{p_S}{q_{S/I}} - 1\right)}\, r \left[\frac{\Delta\beta}{\beta} - \frac{\Delta\gamma}{\gamma}\right], \tag{7}$$

which is the equation given in the main text.

S4 The role of host immunity: SIRS model

We now extend the model of section S3 by assuming that infected individuals become immune (R) when they recover. Loss of immunity ($R \to S$) occurs at rate ρ The per-capita growth rate of individuals of type $i = I$ or J takes the same form as previously

$$\lambda_i = (1-P)\beta_i q_{S/i} + P\sigma\beta_i p_S - \gamma_i.$$

If follows that, in a monomorphic population, equation (4) still holds true in this model. From the expression for invasion fitness λ_J, we find that the selection gradient in the SIRS model also follows equation (6).

Now, in order to describe the invasion dynamics in more detail, we need to track four pair densities p_{SJ}, p_{IJ}, p_{JJ} and p_{RJ}. We shall restrict my attention to the limiting case $P = 0$. In this case we obtain the following invasion matrix

$$\boldsymbol{M} = \begin{pmatrix} -\beta_J(\phi + \bar{\phi}q_{M/SJ} - \bar{\phi}q_{o/\underline{S}J}) - \beta_I\bar{\phi}q_{R/SJ} - \gamma_J & 0 & 0 & \rho \\ (\beta_J + \beta_I)\bar{\phi}q_{R/SJ} & -\gamma_I - \gamma_J & 0 & 0 \\ 2\beta_J(\phi + \bar{\phi}q_{M/SJ}) & 0 & -2\gamma_J & 0 \\ \beta_J\bar{\phi}q_{u/SJ} & \gamma_I & \gamma_J & -\rho - \gamma_J \end{pmatrix}.$$

As previously, we can compute the eigenvectors of the neutral invasion matrix associated with eigenvalue 0 and we find

$$\boldsymbol{v} = \begin{pmatrix} 2\frac{\gamma+\rho}{\rho} & 1 & 1 & 2 \end{pmatrix}$$

and

$$\boldsymbol{u} = \begin{pmatrix} q_{S/J} & q_{I/J} & q_{J/J} & q_{R/J} \end{pmatrix}^T = \begin{pmatrix} \frac{\gamma}{\beta} & \bar{\phi}q_{I/SJ} & \phi + \bar{\phi}q_{J/SJ} & \frac{\gamma}{\gamma+\rho}(1 - \bar{\phi}q_{S/SJ}) \end{pmatrix}^T.$$

Using the perturbation analysis of section S3, we find

$$\begin{aligned} \Delta\lambda_J =& \Delta\beta\, q_{S/J}[2(\gamma+\rho)\bar{\phi}q_{S/SJ} + \rho\bar{\phi}q_{I/SJ} + 2\rho\bar{\phi}q_{R/SJ} - 2\gamma(\phi + \bar{\phi}q_{J/SJ})] \\ & - \Delta\gamma[2(\gamma+\rho)q_{S/J} + \rho(q_{I/J} + 2q_{R/J})] \end{aligned} \tag{8}$$

after neglecting the terms in $\Delta q_{i/SJ}$ with $i = S, I, J, R$. We can simplify things further by noting that, from the expression of $\boldsymbol{u}$, we have $q_{S/J} = \gamma/\beta$, $\bar{\phi}q_{I/SJ} = q_{I/J}$, $\phi + \bar{\phi}q_{J/SJ} = q_{J/J}$, $\bar{\phi}q_{S/SJ} = 1 - (\gamma+\rho)q_{R/J}/\gamma$. Furthermore, we have $q_{I/J} = 1 - q_{S/J} - q_{R/J} - q_{J/J} = 1 - q_{S/I} - q_{R/J} - q_{J/J}$. Assuming that, in the neutral model, $q_{R/J} \approx q_{R/I}$ and $q_{R/SJ} \approx q_{R/SI}$, we are left with the task of computing the equilibrium density of $q_{R/SI}$ in the monomorphic model. I show below that this yields an implicit expression in function of $q_{R/I}$. After some rearrangements and simplifications, we obtain

$$\beta\Delta q_{S/J} = -\frac{\gamma}{1 + q_{R/I} + (u+1)q_{S/I}}\left[\Big(r + u(r - q_{I/I} + q_{I/R})\Big)\frac{\Delta\beta}{\beta} - r\frac{\Delta\gamma}{\gamma}\right], \tag{9}$$

which yields the equation given in the main text. Combining equations (6) and (9), we find that the ESS in a viscous population ($P = 0$) is the solution of

$$\frac{(\Delta\beta/\beta)}{(\Delta\gamma/\gamma)} = \frac{1 + q_{R/I} + (u+1)q_{S/I} - r}{1 + q_{R/I} + (u+1)q_{S/I}) - r + u(q_{I/I} - q_{I/R} - r)}, \tag{10}$$

where $u = 2d/\rho$. Extensive simulations suggest that $q_{I/I} - q_{I/R} < \phi$ (results not shown), and because $r > \phi$ (Lion and Gandon, 2009; Lion, 2009), we find that the right-hand side of equation (10) is greater than 1. For a concave-down trade-off between β and γ, this implies that the ESS for $P = 0$ is lower than the ESS for $P = 1$.

The equlibrium value of $q_{R/SI}$ in the monomorphic population remains to be calculated. The starting point is the equation for the dynamics of p_{SR} pairs. At equilibrium, this gives

$$\gamma p_{SI} + \rho p_{RR} - (\rho + \beta\bar{\phi}q_{I/SR})p_{SR} = 0.$$

Using the fact that $q_{I/SR}p_{SR} = q_{R/SI}p_{SI}$, $q_{R/I}p_I = q_{I/R}p_R$, and $q_{S/I} = \gamma/\beta$, we obtain

$$\bar{\phi}q_{R/SI} = q_{S/I} + \frac{\rho}{\gamma}(q_{R/R} - q_{S/R})\frac{p_R}{p_I}. \tag{11}$$

Now, the dynamics of RR pairs is simply

$$\frac{dp_{RR}}{dt} = \gamma p_{RI} - \rho p_{RR},$$

which gives at equilibrium

$$q_{R/R} = \frac{\gamma}{\rho} q_{I/R} \tag{12}$$

Furthermore, the dynamics of the global density of R sites is

$$\frac{dp_R}{dt} = \gamma p_I - \rho p_R,$$

so we have at equilibrium, using the fact that $q_{I/R} p_R = q_{R/I} p_I$

$$\frac{p_I}{p_R} = \frac{\rho}{\gamma} = \frac{q_{I/R}}{q_{R/I}}. \tag{13}$$

It follows from equation (12) and (13) that

$$q_{R/R} = q_{R/I} \tag{14}$$

Putting equations (11)-(14) together, we obtain

$$\bar{\phi} q_{R/SI} = q_{S/I} + q_{R/I} - q_{S/R},$$

and because $q_{S/R} + q_{R/R} + q_{I/R} = 1$, we have finally, using equations (12) and (13) once more

$$\bar{\phi} q_{R/SI} = q_{S/I} - 1 + \frac{2\gamma + \rho}{\gamma} q_{R/I},$$

which can be used in equation (8) to obtain equation (10).

S5 The role of host demography: oSI model

In this section, we extend the SIS model studied in section S3 by taking into account host demography. Susceptible hosts can reproduce into neighbouring empty sites at rate b, or die at rate d. Infected individuals die at rate $d + \alpha$ where α represents disease-induced mortality (i.e. virulence). The traits affected by host exploitation are β and α. The strategy of the resident parasite yields rates β_I and α_I, and the traits of individuals infected by a mutant parasite are β_J and α_J.

Again, the dynamics of the vector of pair densities $\boldsymbol{p} = \begin{pmatrix} p_{oJ} & p_{SJ} & p_{IJ} & p_{JJ} \end{pmatrix}^T$ is given by

$$\frac{d\boldsymbol{p}}{dt} = \boldsymbol{M}\boldsymbol{p},$$

where the invasion matrix $\boldsymbol{M}$ is

$$\boldsymbol{M} = \begin{pmatrix} -\bar{\phi} b q_{S/oJ} - (d+\alpha_J) & d + (1-P)\beta_J \bar{\phi} q_{o/SJ} + P\beta_J p_S \frac{q_{o/S}}{q_{S/J}} & d+\alpha_I & d+\alpha_J \\ \bar{\phi} b q_{S/oJ} & X & 0 & 0 \\ 0 & (1-P)(\beta_I+\beta_J)\bar{\phi} q_{I/SJ} + P\beta_I p_I + P\beta_J p_I \frac{q_{S/I}}{q_{S/J}} & -2d-\alpha_I-\alpha_J & 0 \\ 0 & 2\beta_J(Pp_J + (1-P)(\phi + \bar{\phi} q_{J/SJ})) & 0 & -2(d+\alpha_J) \end{pmatrix},$$

with

$$X = -2d - \alpha_J - P\beta_I p_I - (1-P)\beta_I \bar{\phi} q_{I/SJ} - (1-P)\beta_J(\phi + \bar{\phi} q_{J/SJ} - \bar{\phi} q_{S/SJ}) - P\beta_J p_J + P\beta_J p_S \frac{q_{S/S}}{q_{S/J}}.$$

As in sections S3 and S4, we compute the left and right eigenvectors of the invasion matrix associated with eigenvalue 0 in the neutral model, and find

$$\boldsymbol{v} = \begin{pmatrix} 2 & v_S & 1 & 1 \end{pmatrix},$$

where v_S has a complicated expression, and

$$\boldsymbol{u} = \begin{pmatrix} q_{o/I} & q_{S/I} & q_{I/J} & q_{J/J} \end{pmatrix}^T.$$

With some tedious algebra, we can compute the first-order effect of selection on the invasion matrix, and solve for $\Delta q_{S/J}$ exactly as we did previously. Neglecting all terms of the form $\Delta q_{x/yz}$, we obtain

$$\Delta q_{S/J} = \frac{\Delta q}{K},$$

where

$$K = \beta[Pp_S(2q_{o/S} + v_S q_{S/S}) + q_{S/I}(Pp_I + (1-P)\boldsymbol{v}.\boldsymbol{u})]$$

and

$$\Delta q = q_{S/I}[\Delta\alpha q_{J/J} - \Delta\beta F_\beta].$$

The calculation above readily gives the following expression for F_β

$$F_\beta = \frac{d+\alpha}{\beta}[q_{J/J} + (v_S - 2)(q_{J/J} - \bar{\phi} q_{S/SI} + q_{S/I} + Pp_S(\bar{\phi} q_{S/SI} - q_{S/S}))],$$

which gives conditions (8) and (10) in the main text with the notations $u = v_S - 2$ and $r = q_{J/J}$. Under a concave-down trade-off, this is enough to predict under which conditions host exploitation should evolve to a lower level than predicted by non-spatial theory, but to compute numerically the ESS, we need some expressions for $\boldsymbol{v}.\boldsymbol{u}$, v_S, and r. We have

$$\boldsymbol{v}.\boldsymbol{u} = 2q_{o/I} + v_S q_{S/I} + q_{I/J} + q_{J/J},$$

and v_S can be computed from the neutral matrix. From the neutral pair dynamics, relatedness is computed using pair approximation as

$$r = q_{J/J} = \phi(1-P)\frac{\beta q_{S/I}}{d+\alpha}.$$

Hence, $r = \phi$ when dispersal is local ($P = 0$), which is an underestimate of the "true" value, but this approximation performs fairly well for higher values of P.

To sum up, we have fully expressed the selection gradient in terms of quantities that need to computed in a monomorphic population with the resident traits at equilibrium, and we can numerically integrate the pair dynamics (using standard pair approximation) to find these equilibrium values. By repeating this procedure, we can find the ESS. This is how we produced the plain curve in figure 1b in the main text.

References

Boots M. and Sasaki A. (1999) 'Small worlds' and the evolution of virulence: infection occurs locally and at a distance. *Proc. Roy. Soc. Lond. B.* **266**: 1933–1938.

Ferrière R. and Le Galliard J.-F. (2001) Invasion fitness and adaptive dynamics in spatial population models. In Clobert J., Danchin E, Dhondt A. A., and Nichols J. D. eds., *Dispersal.* Oxford University Press.

Lion S. (2009) Relatedness in spatially structured populations with empty sites: an approach based on spatial moment equations. *J. theor. Biol.* **260**(1): 121–131.

Lion S. and Gandon S. (2009) Habitat saturation and the spatial evolutionary ecology of altruism. *J. of evol. Biol.* **22**: 1487–1502.

Lion S. and van Baalen M. (2009) The evolution of juvenile-adult interactions in populations structured in age and space. *Theor. Popul. Biol.* **76**(2): 132–145.

Rand D. A. (1999) Correlation equations and pair approximation for spatial ecologies. In McGlade J. ed., *Advanced ecological theory*, pp. 100–142. Blackwell, Oxford.

van Baalen M. and Rand D. A. (1998) The Unit of Selection in Viscous Populations and the Evolution of Altruism. *J. theor. Biol.* **193**: 631–648.
